# Chromosome 2q gain and epigenetic silencing of GATA3 in microglandular adenosis of the breast

**DOI:** 10.1002/cjp2.195

**Published:** 2020-12-31

**Authors:** Martin Radner, Jana Lisa van Luttikhuizen, Stephan Bartels, Janin Bublitz, Isabel Grote, Luisa Rieger, Henriette Christgen, Helge Stark, Christopher Werlein, Marcel Lafos, Doris Steinemann, Ulrich Lehmann, Matthias Christgen, Hans Kreipe

**Affiliations:** ^1^ Institute of Pathology Hannover Medical School Hannover Germany; ^2^ Department of Human Genetics Hannover Medical School Hannover Germany

**Keywords:** breast cancer precursor, triple‐negative breast cancer, luminal differentiation, stem cell, DNA methylation, epigenetic alteration

## Abstract

Microglandular adenosis (MGA) represents a rare neoplasm of the mammary gland, which in a subset of cases may be associated with triple‐negative breast cancer (BC). The biology of MGA is poorly understood. In this study, eight MGA cases (*n* = 4 with and *n* = 4 without associated BC) were subjected to a comprehensive characterization using immunohistochemistry, genome‐wide DNA copy number (CN) profiling, fluorescence *in situ* hybridization (FISH), next‐generation sequencing (NGS), and DNA methylation profiling using 850 K arrays and bisulfite pyrosequencing. Median patient age was 61 years (range 57–76 years). MGA lesions were estrogen receptor (ER)‐negative, progesterone receptor‐negative, HER2‐negative, and S100‐positive. DNA CN alterations (CNAs) were complex or limited to few gains and losses. CN gain on chromosome 2q was the most common CNA and was validated by FISH in five of eight cases. NGS demonstrated an average of two mutations per case (range 0–5) affecting 10 different genes (*ARID1A*, *ATM*, *CTNNB1*, *FBXW7*, *FGFR2*, *MET*, *PIK3CA*, *PMS2*, *PTEN*, and *TP53*). CNAs and mutations were similar in MGA and adjacent BC, indicating clonal relatedness. DNA methylation profiling identified aberrant hypermethylation of CpG sites within *GATA3*, a key transcription factor required for luminal differentiation. Immunohistochemistry showed regular GATA3 protein expression in the normal mammary epithelium and in ER‐positive BC. Conversely, GATA3 was reduced or lost in all MGA cases tested (8/8). In conclusion, MGA is characterized by common CN gain on chromosome 2q and loss of GATA3. Epigenetic inactivation of GATA3 may provide a new clue to the peculiar biology of this rare neoplasia.

## Introduction

Microglandular adenosis (MGA) of the mammary gland was first described in 1968 and was initially considered a benign lesion [1]. MGA may present as a mass‐forming, palpable tumor or as an incidental microscopic finding in breast biopsies [2]. Histologically, it is characterized by small, round tubules haphazardly spread in the mammary fat tissue, without desmoplastic reaction [2, 3, 4, 5, 6]. Nuclear atypia is absent or mild, as is mitotic activity.

Immunohistochemically, MGA shows a peculiar phenotype characterized by the absence of estrogen receptor (ER) and progesterone receptor (PR) expression but positive staining for S100 [3]. MGA also lacks overexpression of HER2 and is thus a triple‐negative lesion [5]. Furthermore, MGA lacks a myoepithelial cell layer surrounding the haphazardly spread tubules. Accordingly, MGA is also negative for myoepithelial or basal differentiation markers, including CK5/6 and p63 [3, 5]. Strong immunoreactivity for S100 remains a key diagnostic feature, and this feature was discovered as an empirical finding [3]. The biology of MGA is still poorly understood.

After the initial descriptions, invasive breast cancer (BC) was found to subsequently develop years after the diagnosis of MGA [2, 7]. In a subset of MGA cases, synchronous adjacent invasive BC may be encountered. These invasive BCs tend to share some morphological features with MGA, such as cytoplasmic clearing and a triple‐negative immunophenotype [5]. Cases of MGA with more pronounced nuclear atypia (occasionally termed atypical MGA, aMGA) may represent lesions in transition from MGA to BC [3].

So far, only a very limited number of molecular studies on MGA has been reported [8, 9, 10, 11, 12]. The total number of MGA cases included in the studies cited above is 38 cases (in total) [8, 9, 10, 11, 12]. Using high‐resolution comparative genomic hybridization, Shin *et al* have demonstrated variable and partially complex copy number (CN) alterations (CNAs) in MGA [9]. Recurrent CNAs included gain of chromosome 2q, which was detected in 4 of 12 (33%) cases [9]. In a similar study, using CN estimates from targeted‐capture, massively parallel sequencing data, Geyer *et al* described complex CNAs in MGA and aMGA [12]. Recurrent CNAs included gains on chromosome 2q, which were detected in 6 of 10 cases (60%) [12]. In a related study by Guerini‐Rocco *et al*, gains on chromosome 2q were detected in 7 of 10 (70%) cases of MGA with adjacent invasive triple‐negative BC, but not in MGA without BC (0/2 cases) [11]. MGA and adjacent invasive BC showed similar CNAs, indicating that MGA was a precursor lesion of triple‐negative BC in these patients [11].

Using next‐generation sequencing (NGS), Guerini‐Rocco *et al* also studied MGA for somatic mutations in 236 genes known to be recurrently mutated in BC [11]. *TP53* was shown to be the sole highly recurrent gene mutation in MGA. *TP53* was affected in six of seven (86%) cases of MGA with adjacent invasive triple‐negative BC, but not in MGA without BC (0/2 cases) [11]. The overall repertoire of genetic alterations in MGA resembled that of triple‐negative BC [10]. Guerini‐Rocco *et al* concluded that MGA belongs to a spectrum of rare, low‐grade, triple‐negative neoplasms of the mammary gland [10]. To date, MGA remains an enigmatic lesion; while the histomorphological appearance strongly suggests luminal differentiation, the molecular features are similar to triple‐negative BC [11].

In this study, eight cases of MGA, including four lesions without adjacent invasive BC, were analyzed. NGS was applied for mutational profiling, and molecular inversion probe (MIP) array analysis was used for high‐resolution genome‐wide DNA CN profiling. Unlike previous studies, DNA methylation patterns were also assessed and included in the molecular characterization of MGA.

## Materials and methods

### Tissue specimens

Formalin‐fixed paraffin‐embedded (FFPE) specimens of MGA were retrieved from the archives of the Institute of Pathology at the Hannover Medical School (MHH). Most cases (7/8 cases) were submitted to the MHH from other institutes for histopathological consultation, and final reference diagnosis was made by Prof. Hans Kreipe (HK). This study was conducted in accordance with the guidelines of the local ethics committee (Hannover Medical School, Hannover, Germany).

### Immunohistochemistry

For immunohistochemistry, 1 μm‐thick sections of FFPE tissue blocks were mounted on superfrost slides (Thermo Fisher Scientific, Rockford, IL, USA). Next, slides were deparaffinized and rehydrated conventionally and were subjected to immunohistochemical staining on a Benchmark Ultra (Ventana, Tucson, AZ, USA) automated stainer. The CC1 mild program was used for antigen retrieval, and the ultraView DAB kit (Ventana) was used for signal detection. Antibodies and scoring methods used for immunohistochemistry are summarized in supplementary material, Table S1.

### DNA extraction

Genomic DNA was extracted as described previously [13]. In brief, MGA or BC tissue or normal mammary gland tissue was marked on hematoxylin and eosin (HE)‐stained sections of FFPE tissue blocks. Corresponding tissue areas were macrodissected with a surgical blade on unstained sections (*n* = 10, 8 μm each) from corresponding FFPE blocks. Another HE stain, prepared after cutting off the unstained sections, confirmed unaltered tissue representation on deeper sections of the blocks. Next, genomic DNA and RNA were extracted with the Maxwell RSC DNA and RNA FFPE kits (Promega, Madison, WI,USA) using a Maxwell RSC instrument (Promega) according to the manufacturer's recommendations. DNA was quantified using a Qubit 2.0 fluorometer (Invitrogen, Darmstadt, Germany) and the Qubit dsDNA HS assay kit (Life Technologies, Carlsbad, CA, USA).

### DNA CN profiling

Whole‐genome DNA CN profiling was performed using MIP arrays (OncoScan™; Affymetrix, Santa Clara, CA, USA) and approximately 80 ng total DNA, as described previously [13]. OSCHP files were produced from CEL files by Chromosome Analyses Suite (ChAS) software (version 4.0.0.385, applied biosystems by Thermo Fisher Scientific, Waltham, MA, USA). CNAs were detected using the TuScan algorithm. The criteria for CN gains were ≥50 markers and ≥50 kb size. The criteria for CN losses were ≥25 markers, and regions of loss of heterozygosity had to be ≥3000 kb in size. Four samples showed highly complex CNAs, complicating manual recentering of CN profiles and detailed analyses. For MGA case 2, the CN profile was recentered manually based on the data corresponding to the adjacent BC and the allele difference, B‐allele frequency, and weighted log2 ratio of chromosome 21. Of several quality control metrics, mean absolute percentage deviation (MAPD) and single nucleotide polymorphism (SNP) quality control of normal diploid markers (ndSNPQC) are the two pivotal metrics that measure the noisiness of log2 ratios. The MGA of case 1 and its adjacent BC showed critical quality control parameters (MAPD ≤0.3 and ndSNPQC ≥26), which were attributed to the high number of CNAs in these specimens. Weighted log2 ratios of all samples were extracted from the ChAS software and were subsequently analyzed with the R package ‘Clonality’ (version 1.26.0) [13, 14, 15]. The clonal relatedness of CN profiles was determined using the likelihood ratio (LR) method. The LR quantifies the odds that two given lesions are clonal and is benchmarked against the distribution of LRs in pairs of independent tumors from independent patients in a reference cohort [14]. The MIP array data series is deposited in the Gene Expression Omnibus (GEO) database (GSE141831).

### Next‐generation sequencing

Mutational analysis was carried out by NGS as described previously [16]. NGS was performed with genomic DNA on an Ion S5 system (Life Technologies) using a commercial NGS panel (Oncomine Comprehensive Assay v3) covering 161 genes (mutations, indels, CNA, and gene fusions) according to the manufacturer's recommendations (see supplementary material, Table S2).

### Fluorescence *in situ* hybridization

For fluorescence *in situ* hybridization (FISH), 4 μm‐thick sections of FFPE tissue blocks were mounted on superfrost slides (Thermo Fisher Scientific). Next, slides were deparaffinized and rehydrated conventionally and were permeabilized and denatured in a microwave oven for 30 min with sodium citrate buffer (pH 6.0) (Sigma Aldrich, St. Louis, MO, USA). Then, slides were cooled to room temperature, rinsed in distilled water, and were incubated in a pepsin solution for 15 min at 37 °C. Subsequently, slides were passed through ascending concentrations of ethanol (70, 80, and 100%) and were hybridized with a commercial FISH probe for the *ERBB4* gene locus on chromosome 2q34 and the AFF3 gene locus on 2q11 close to the centromeric site of chromosome 2 (ZytoLight SPEC ERBB4/2q11 Dual Color Probe; Zytovision, Bremerhaven, Germany). *ERBB4* signals were quantified in 30 tumor cells each. The average *ERBB4* CN per cell was calculated as the sum of the counts of signals divided by 30. The threshold used for the definition of an *ERBB4* CN gain was CN ≥3.0, which is a common threshold for CN gain in diagnostic FISH applications [17].

### DNA methylation profiling by Infinium EPIC array analysis

DNA methylation profiling was performed with genomic DNA extracted from three MGA lesions with sufficient amount of DNA (cases 1, 4, and 5) and an additional nine specimens, including normal mammary gland tissue, unrelated triple‐negative BCs, and ER*‐*positive/HER‐negative BCs, for comparison (see supplementary material, Table S3). The aim of this experiment was to identify the defining features of MGA compared to common BCs. Therefore, independent BCs were considered more appropriate than MGA‐associated invasive BCs for comparison. A total of 400–915 ng total DNA was used from each sample. Following bisulfite treatment and whole‐genome amplification, DNA products were denatured and hybridized to Infinium Human Methylation EPIC 850 K bead chips (Illumina, San Diego, CA, USA) according the manufacturer's protocol. The EPIC 850 K array data series is deposited in the GEO database (GSE150654). Array data analysis was performed with R (version 3.4.4). The R package Illumina Human Methylation EPICanno.ilm10b3.hg19 (version 0.6.0) was used for chip annotation. The R package MethylAid (version 1.12.0) was used for quality control [18]. The R package ChAMP (version 2.9.10) was used for data loading (default settings) and statistical analyses, and the BMIQ algorithm was used for intrasample probe normalization [19, 20]. Differentially methylated probes (DMPs, reflecting individual CpG sites) were identified with limma included in ChAMP using a false discovery rate (FDR) threshold of ≤0.1 [21].

### Quantitative methylation analysis by bisulfite pyrosequencing

For validation of DNA methylation characteristics, DNA samples already subjected to DNA methylation analysis by Infinium EPIC 850 K array were analyzed by bisulfite pyrosequencing. In addition, we enlarged the group of hormone receptor‐positive breast carcinomas (*n* = 14), triple‐negative breast carcinomas (*n* = 18), and normal mammary glands (*n* = 13). Clinicopathological characteristics of included carcinomas are summarized in supplementary material, Table S4. These DNA samples (*n* = 45) were treated with sodium bisulfite using the EZ DNA Methylation Kit™ (Zymo Research, Freiburg, Germany) according to the manufacturer's instructions and then eluted in 40 μl of elution buffer. Bisulfite pyrosequencing analysis was performed as previously described [22]. Primer sequences and polymerase chain reaction (PCR) conditions are listed in supplementary material, Table S5. CpG site methylation quantification was performed using the methylation Software PyroQ‐CpG™. The criteria for pyrogram selection were as follows: sufficient peak height of >15 units (arbitrary units for light emission calculated by the software), symmetric peaks without any irregularities or side peaks, wide reading length with a high reliability until the end of the sequence, and absence of any significant signals at the positions where a bisulfite treatment control was included or where control nucleotides were dispensed to check for nonspecific background signals.

## Results

### Clinicopathological characteristics

**Figure 1:**
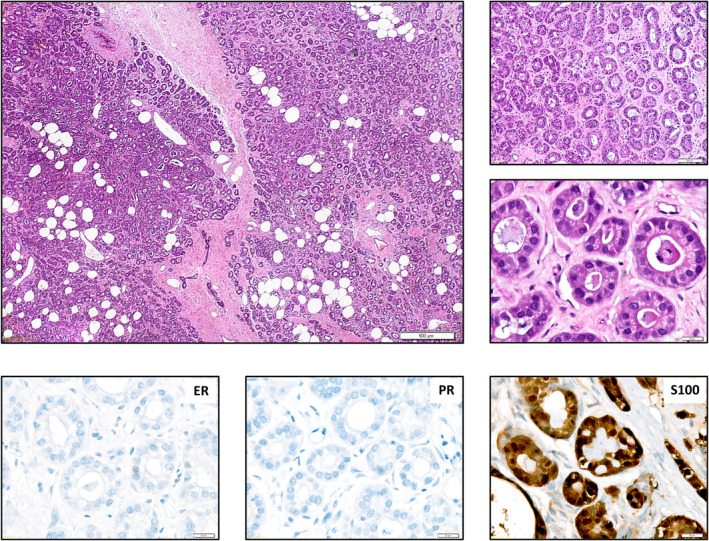
Histomorphology of a representative MGA case with haphazardly arranged glandular formations with minimal nuclear anisomorphy, immunohistochemically positive for S100, and negative for ER and PR (hematoxylin and eosin, immunoperoxidase).

All cases showed the characteristic histology of MGA (Figure 1). Median patient age was 61 years (range 57–76 years) (Table 1). The lesions consisted of small, round glands that were distributed haphazardly in fibrous or fatty tissue. In some of the cases, the glands showed a comparatively dense arrangement (cases 1, 2, 6, and 5) (Figure 1). In others (cases 3, 4, 7, and 8), individual glands were widely scattered. Glands were lined by a single layer of flat or cuboidal epithelial cells and lacked a myoepithelial cell layer. Luminal eosinophilic secretions were frequently noted but were not always evident. The cytoplasm was typically amphophilic, but cytoplasmic clearing could also be observed. Nuclear atypia was mostly minimal or absent. Three cases (cases 2, 5, and 7) displayed slightly more pronounced nuclear atypia, stratification of the epithelial cells, and variation in size and shape of the glands, compatible with aMGA. Four cases (cases 1, 2, 5, and 7) were associated with adjacent synchronous invasive BC. The spatial transition from MGA to the frankly invasive adjacent BC was abrupt in some cases (cases 1 and 2) and gradual or ill‐defined in other cases (cases 5 and 7). Lesional cells were strongly positive for S100 and completely lacked ER and PR expression (Table 2 and supplementary material, Figure S1). Only case 4 showed weak expression of PR in approximately 20% of lesional cells. Androgen receptor was negative in all cases. There was no overexpression of HER2. A myoepithelial cell layer was absent in all cases, as determined by immunohistochemistry for CK5/14, p63, and CD10. However, focal weak expression for p63 and CD10 was noted in some lesional cells in cases 4 and 7. Nuclear accumulation of p53 was not observed. Only case 2 showed a mosaic‐like expression of p53 protein in approximately 75% of lesional cells. Proliferative activity, as determined by immunohistochemistry for Ki67, proved to be heterogeneous, and the Ki67 index ranged from 5 to 25% irrespective of the presence or absence of adjacent BC. Adjacent BCs were all triple‐negative and S100‐positive (Table 2).

**Table 1:** Clinicopathological characteristics.

Case	Age	Localization	Histology	Additional lesions
Case 1	71	n.a.	MGA	BC, triple‐negative, mucinous, G2
Case 2	61	n.a.	aMGA	DCIS, BC, triple‐negative, IDC G3
Case 3	59	Right, o/l	MGA	Intraductal papilloma
Case 4	60	Right	MGA	–
Case 5	64	Left, i/u	aMGA	BC, invasive carcinoma reminiscent of ACC, triple‐negative, G1
Case 6	61	Right	MGA	–
Case 7	76	Right, o/u	aMGA	BC, triple‐negative, IDC, G1
Case 8	57	Left	MGA	–

ACC, acinic cell carcinoma; DCIS, ductal carcinoma *in situ*; i/u, inner/upper; IDC, invasive ductal carcinoma; o/l, outer/lower; o/u, outer/upper.

**Table 2:** Immunohistochemical characteristics.

Case	ER	PR	AR	HER2	MGBN	CK5/14	p63	CD10	EMA	CK7	CK8/18	S100	CD117	P53	SOX10	Ki67
**MGA**																
Case 1	0	0	0	0	5 (2)	0	0	0	60 (2)	100 (3)	100 (3)	100 (3)	30 (1)	0	90 (1)	5
Case 2	0	0	0	5 (1)	5 (3)	0	0	0	40 (2)	100 (3)	100 (3)	75 (3)	5 (1)	75 (3)	5 (1)	5
Case 3	0	0	0	0	5 (2)	0	0	0	30 (1)	95 (2)	0	100 (3)	5 (1)	0	0 (0)	0
Case 4	0	20 (1)	0	0	5 (2)	0	0	5 (1)	0	100 (3)	100 (2)	70 (3)	100 (3)	5 (1)	80 (1)	25
Case 5	0	0	0	0	0	0	0	0	15 (1)	100 (3)	100 (2)	100 (3)	0	0	70 (1)	10
Case 6	0	0	0	0	0	0	0	0	0	80 (2)	0	100 (3)	80 (3)	0	0 (0)	25
Case 7	0	0	0	0	70 (1)	0	30 (1)	0	0	100 (3)	95 (2)	85 (3)	90 (1)	0	60 (1)	20
Case 8	0	0	0	0	0	0	0	0	0	100 (3)	100 (3)	100 (3)	95 (2)	5 (1)	75 (1)	5
**Adjacent BC**																
Case 1	0	0	0	0	5 (1)	0	0	0	75 (3)	35 (2)	95 (2)	100 (3)	5 (2)	0	90 (1)	25
Case 2	0	0	0	5 (1)	5 (3)	0	0	0	40 (2)	90 (3)	90 (2)	65 (3)	5 (1)	100 (3)	85 (1)	40
Case 5	0	0	0	0	0	0	0	0	15 (1)	100 (3)	100 (2)	100 (2)	0	5 (1)	85 (1)	10
Case 7	0	0	0	0	70 (1)	0	30 (1)	0	0	100(3)	95 (2)	85 (3)	90 (1)	0	70 (1)	20

Percentage of positive lesional cells. Staining intensity is given in parenthesis: 1 (weak), 2 (moderate), 3 (strong).

AR, androgen receptor; EMA, epithelial membrane antigen; MGBN, mammaglobin.

### Complex CNA and common gain of chromosome 2q

**Figure 2:**
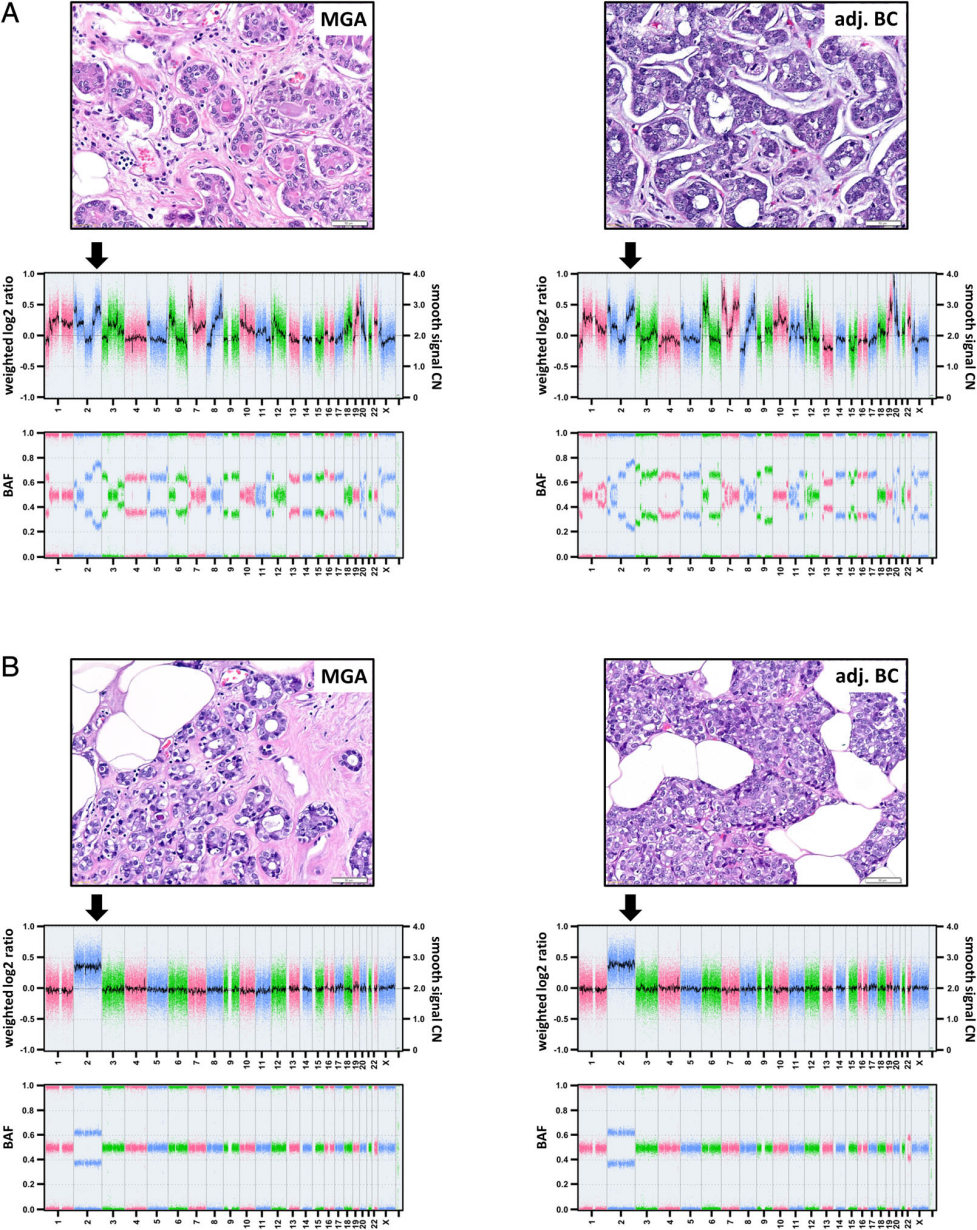
Whole‐genome CNA profiles of cases (A) 2 and (B) 5. MGA proliferations are depicted on the left side, and adjacent invasive carcinomas are shown on the right side with corresponding histology (hematoxylin and eosin) and CNA profiles. The upper plots show weighted log2 ratios and CNs (represented as a Gaussian‐smoothed calibrated CN estimate) on the left and right *y*‐axes, respectively. Chromosomal localization is represented on the *x*‐axis. The lower plots show the corresponding B‐allele frequency (BAF). Gains of chromosome 2q can be seen in both MGA cases and the adjacent carcinomas.

**Figure 3:**
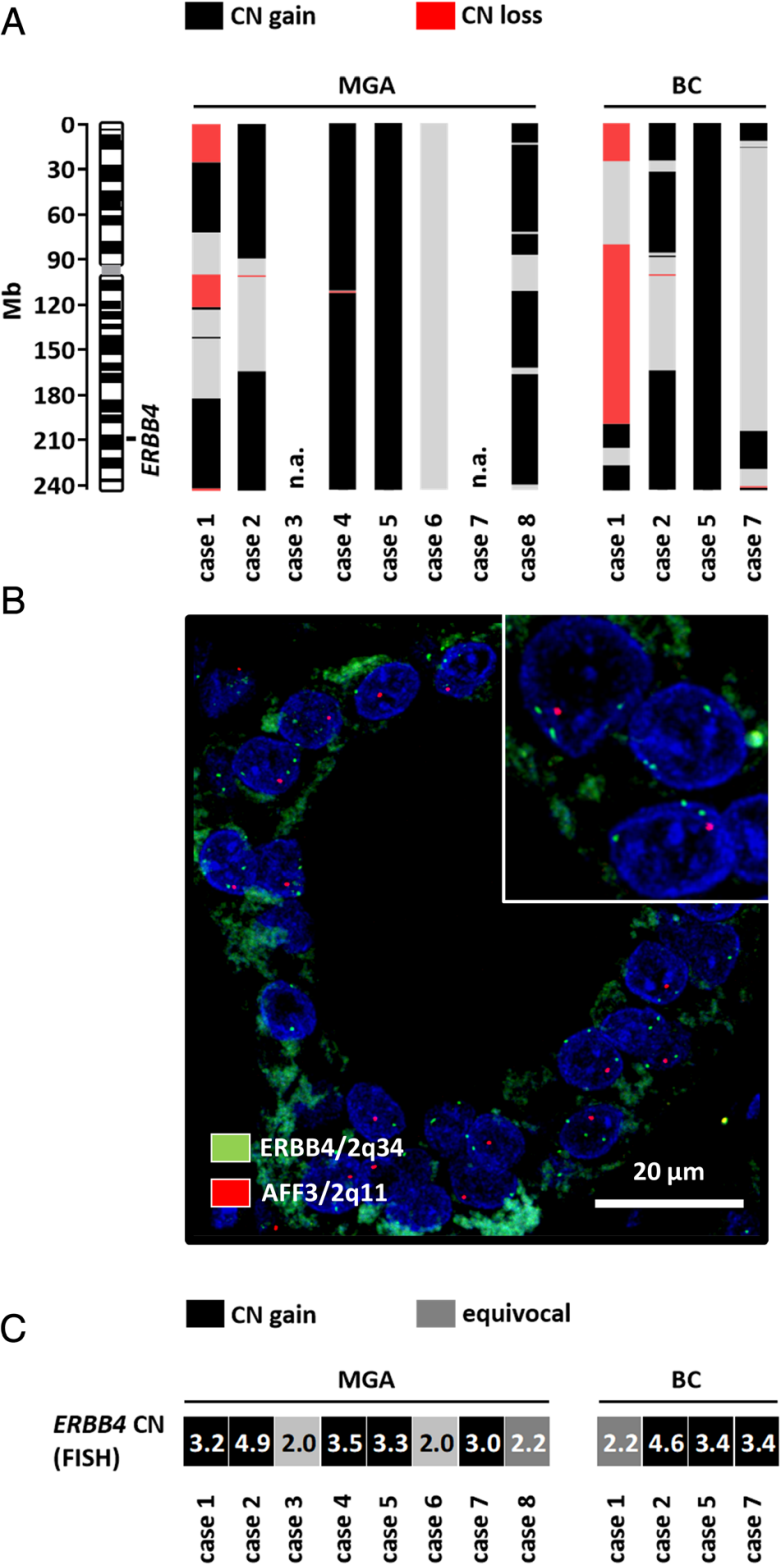
Simplified depiction of CN gain of (A) chromosome 2 and (B and C) FISH results. As can be seen from the black bars spanning region 2q with varying extensions, all cases except case 6, including adjacent carcinomas, share overlapping gains in this chromosomal region. A representative FISH result is shown in (B), with low level increase of green *ERBB4* signals on chromosome 2q34 (case 1). The average CN of *ERBB4* CN (chromosome 2q34) as detected by FISH is detailed in (C).

Microdissected MGA lesions were subjected to genome‐wide DNA CN profiling using the MIP array technology. Adequate CN profiles were obtained from six of eight MGA cases. In two cases (cases 3 and 7), CN profile could not be obtained due to insufficient DNA amount or quality. Overall, CN profiles showed variable characteristics (see supplementary material, Table S6). Highly complex patterns of CNAs were observed in two of six cases (cases 1 and 2) (Figure 2A). Comparatively simple patterns of CNAs were observed in four of six cases (cases 4, 5, 6, and 8) (Figure 2B). CN gain on chromosome 2 was the most common alteration identified (Figure 3A). Only case 6 did not show a gain on chromosome 2 (Figure 3A and supplementary material, Figure S2).

The microdissected tissue of adjacent triple‐negative BCs was also subjected to DNA CN profiling. Adequate CN profiles were obtained from a total of four adjacent BCs (cases 1, 2, 5, and 7). In three cases (cases 1, 2, and 5), CN profiles from both lesions (MGA and invasive BC) were obtained. CNAs of MGA and adjacent BC were highly similar, indicating clonal relatedness (Figure 2). To analyze clonal relatedness more comprehensively, we employed the statistical LR method [14, 15]. The LR method determines clonality or independence of two tumors based on the overall patterns of CNAs [14, 15]. The complete series of CN profiles from MGA lesions and adjacent BCs (*n* = 10 CN profiles) served as a reference cohort, providing 42 nonclonal lesion pairs from independent patients for this statistical analysis. LR values, reflecting the odds that two lesions are clonal, ranged from 1.7 × 10^−8^ to 5.2 × 10^3^ (median 2.9 × 10^−6^) in lesions from independent patients (see supplementary material, Figure S3). LRs for MGAs and adjacent invasive BCs ranged from 1.5 × 10^4^ to 3.0 × 10^29^, which formally proved the clonal relatedness of adjacent lesions in individual patients (3 × *p* < 0.001).

Consistent with CNAs detected in MGA lesions, adjacent BCs also featured CN gains on chromosome 2 (Figure 3A). Considering all CN profiles, the minimal common region with CN gain on chromosome 2q spanned from approximately 205 to 229 Mb and included the *ERBB4* gene (hg19:212240442–213403352). Subsequently, CN gain on chromosome 2q was validated by FISH using a commercially available probe for the *ERBB4* gene locus (chromosome 2q34). CN gain of the *ERBB4* gene locus was validated by FISH in five of eight MGA lesions and in three of four adjacent invasive BCs (Figure 3B,C and supplementary material, Table S7).

### Broad spectrum of gene mutations

**Figure 4:**
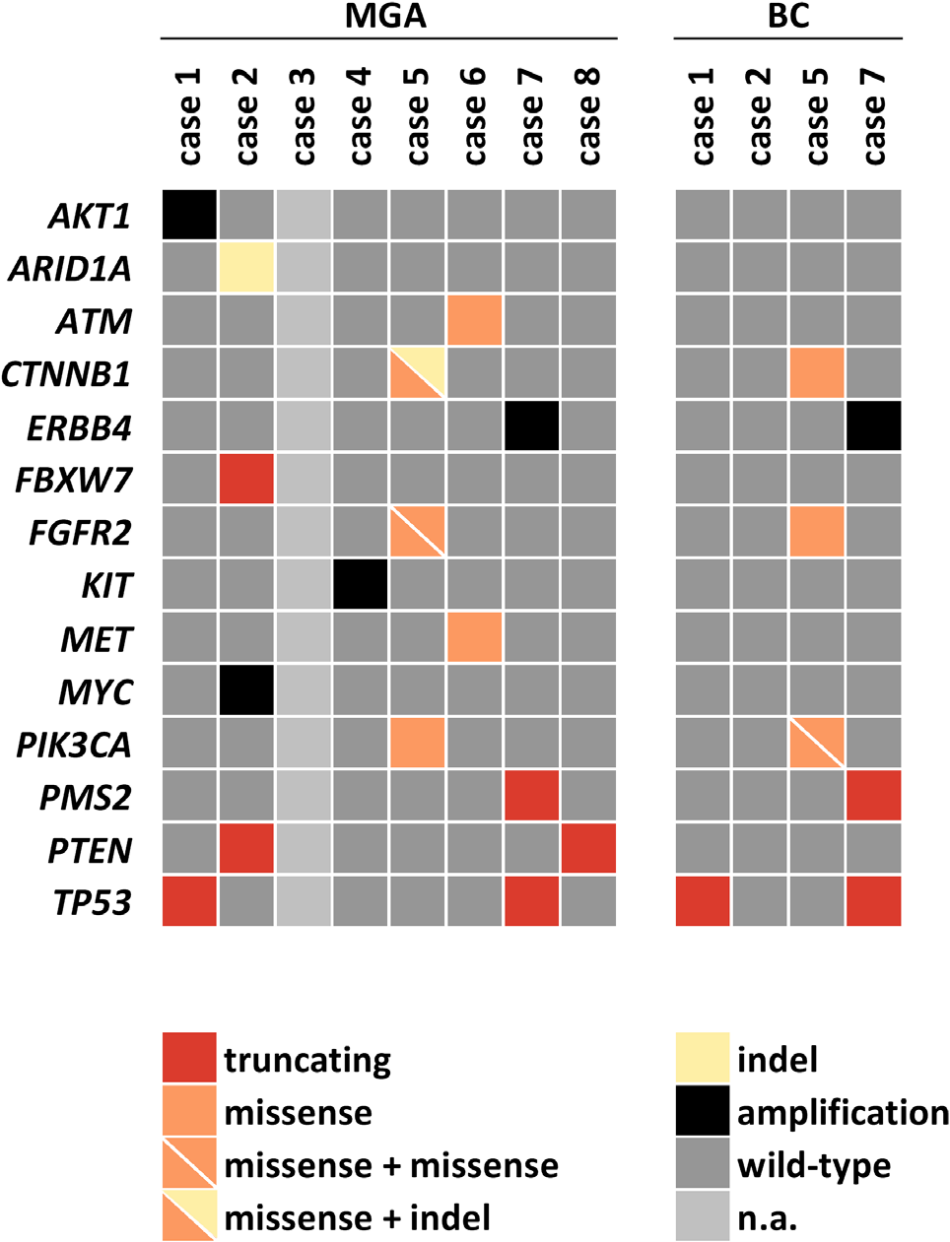
Mutations detected by NGS do not show overlapping aberrations within the genes under study between different MGA cases. MGA and corresponding adjacent carcinomas (BC) revealed identical mutations in individual cases (cases 1, 5, and 7).

Next, microdissected MGA lesions and adjacent invasive BCs were subjected to mutational analysis using a commercially available NGS panel covering mutations and gene fusions of 161 cancer‐related genes (supplementary material, Table S2). Adequate sequencing data were obtained from seven of eight MGA lesions and four adjacent BCs. In one MGA case (case 3), no mutational profile could be obtained due to insufficient DNA quality. NGS identified an average of two mutations per MGA sample (range 0–5), affecting a total of 10 different genes (Figure 4). *TP53* and *PTEN* were the only two genes mutated in more than one MGA. *TP53* mutations were identified in two MGA cases (cases 1 and 7), both of which were associated with invasive BC. Both *TP53* mutations were truncating mutations (p.W146* and p.Q104*) (see supplementary material, Table S8). This correlated well with the complete lack of p53 protein expression in MGA cases 1 and 7, as detected by immunohistochemistry (Table 2). In addition, NGS indicated amplification of four genes, including *ERBB4* (Figure 4). In adjacent BCs, NGS identified an average of 1.7 mutations per BC (range 0–4), affecting a total of five different genes (Figure 4). BCs in cases 1 and 7 featured *TP53* mutations, which were concordant with *TP53* mutations detected in adjacent MGA lesions. The BC in case 5 revealed mutations in *CTNNB1*, *FGFR2*, and *PIK3CA*, which were also concordant with respective mutations in adjacent MGA tissue. However, the BC of case 2 lacked *ARID1A*, *FBXW7*, and *PTEN* mutations that were detected in adjacent MGA. Fusion transcripts were not detected in any case under study. In conclusion, MGA lesions showed a broad spectrum of mutations affecting a variety of different cancer‐related genes. Most of these mutations were also detectable in adjacent invasive BCs, which is consistent with clonal relatedness.

### DNA methylation profiling and hypermethylation of *GATA3*

**Figure 5:**
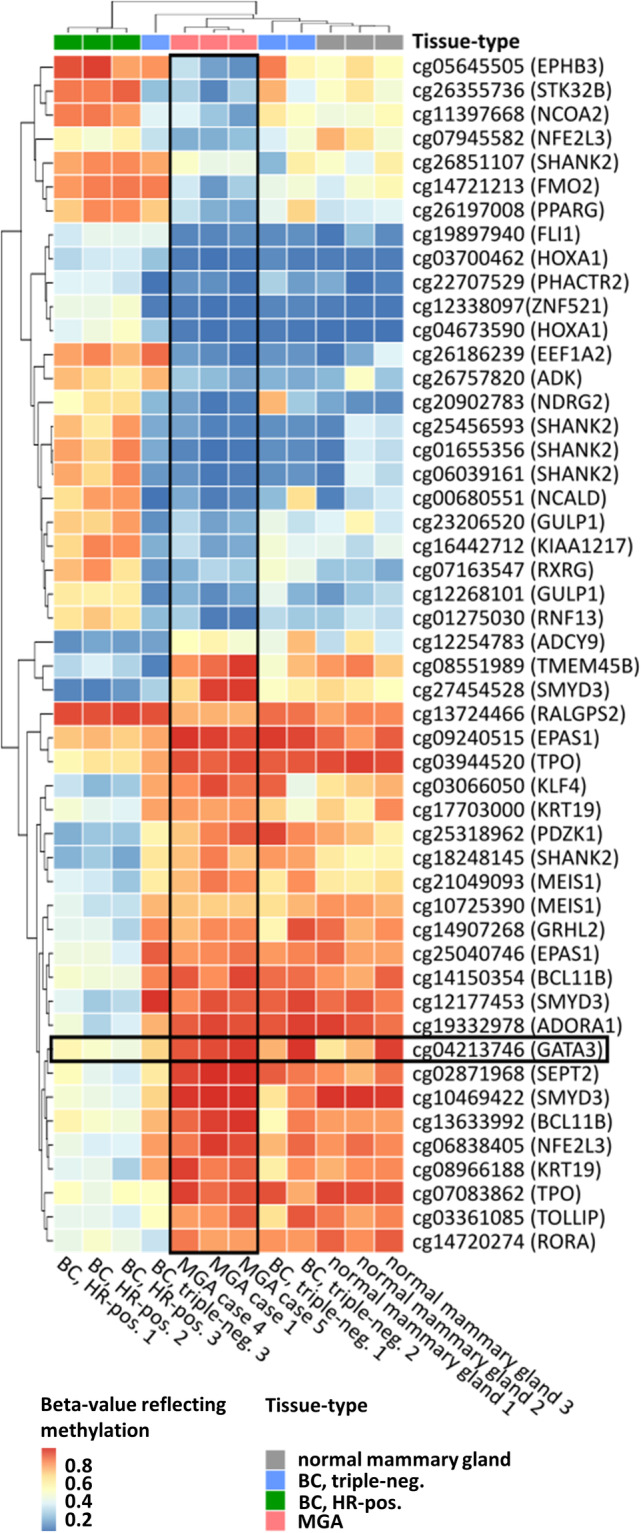
Overall DNA methylation profiles as determined by Illumina EPIC 850 K bead chips demonstrated similar patterns in the three MGA cases studied. Patterns of hyper‐ and hypomethylation were closer to triple‐negative cases and normal mammary gland tissue, whereas luminal (ER‐positive) BCs exhibited divergent patterns of gene methylation.

MGA lesions were highly similar with respect to histomorphology and immunophenotype but showed variable DNA CNAs and mutational characteristics. Because defining molecular alterations may escape CN profiling and mutational analyses when coded epigenetically, we examined genome‐wide DNA methylation patterns in MGA using the Illumina Infinium Methylation EPIC 850 K bead chip technology. Only three of eight MGA lesions (cases 1, 4, and 5) could be included in this exploratory analysis because of the high minimal DNA amount required for this assay. For the identification of differentially methylated positions (DMPs), we also included microdissected normal mammary gland tissue (*n* = 3, unrelated patients), triple‐negative BCs (*n* = 3, unrelated patients), and hormone receptor (HR)‐positive BCs (*n* = 3, unrelated patients) for comparison (see supplementary material, Table S3). Subsequent statistical analyses focused on CpG sites associated with 416 key transcription factors and regulatory genes expressed in the normal mammary gland and in BC, as defined in previous studies (see supplementary material, Table S9) [19, 20]. At an FDR of 0.1, 50 DMPs were identified when comparing MGA lesions with HR‐positive BC (Figure 5). Consistently, aberrant DNA hypermethylation within the *GATA3* gene (CpG site cg04213746) was identified in all three MGA specimens under study (Figure 5 and supplementary material, Table S10). *GATA3* is a critical transcription factor required for luminal differentiation and ER expression in mammary epithelial cells [21, 22, 23, 24].

**Figure 6:**
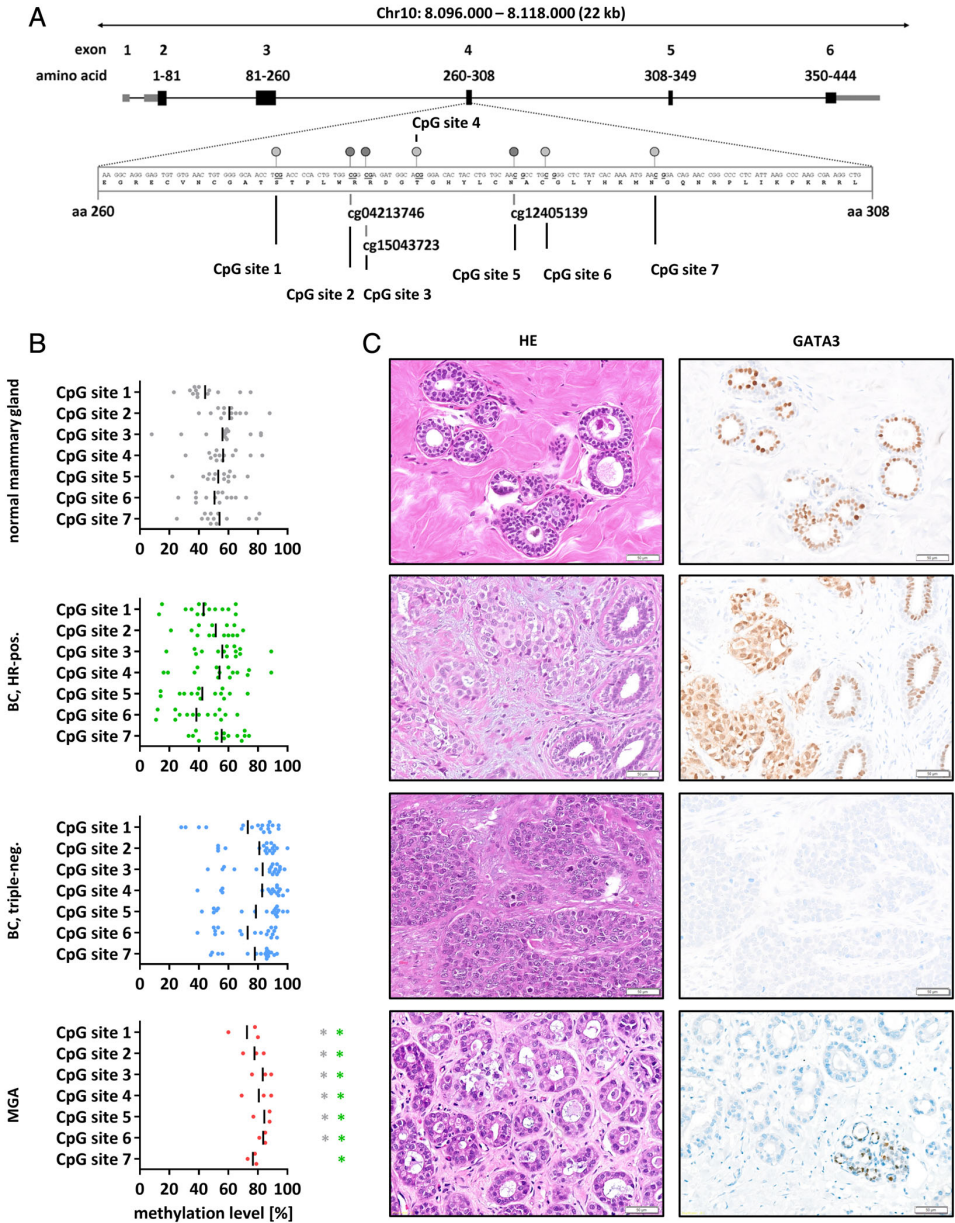
Aberrant methylation of the *GATA3* gene in MGA. The region of the gene with recurrent hypermethylation in MGA is schematically represented in (A). The seven CpG sites within this region exhibited recurrent hypermethylation, discriminating MGA from normal mammary gland and hormone receptor‐positive BC. (B) Triple‐negative BCs resemble MGA and (C) corresponding reduced immunohistochemical GATA3 expression can be seen in both lesions, whereas normal duct epithelium and hormone receptor‐positive cancer demonstrate GATA3 positivity.

To validate aberrant DNA hypermethylation of *GATA3* in MGA, bisulfite pyrosequencing was carried out for seven individual CpG sites in exon 4 of *GATA3*, including cg04213746 (Figure 6A). MGA lesions included three cases (cases 1, 4, and 5). As a control, we included a large collection of normal mammary specimens (*n* = 13), HR‐positive BCs (*n* = 14), and triple‐negative BCs (*n* = 18) (Figure 6B and supplementary material, Figure S4). MGA lesions showed significantly higher DNA methylation levels than normal mammary tissue or HR‐positive BCs for all *GATA3* CpG sites tested (*p* < 0.0437) (Figure 6B). Hence, bisulfite pyrosequencing confirmed aberrant DNA hypermethylation within the *GATA3* gene in MGA.

### Loss of GATA3 protein expression in MGA

Local increases in DNA methylation levels often correlate with transcriptional silencing of affected genes [25]. Because DNA methylation profiling revealed aberrant hypermethylation within *GATA3* in MGA, we hypothesized that GATA3 protein expression is reduced in MGA lesions. Therefore, GATA3 was assessed by immunohistochemistry. In accordance with the molecular findings with regard to *GATA3* gene hypermethylation, immunoreactivity for GATA3 was constantly reduced (cases 2, 4, 5, and 7) or completely missing in all MGA lesions (cases 1, 3, 6, and 8) and adjacent BC (Table 3 and Figure 6C). By contrast, regular GATA3 protein expression occurred in the adjacent normal mammary epithelium of MGA specimens and in ER‐positive BC control samples (Figure 6C and supplementary material, Table S3 and Figure S5).

**Table 3:** Immunohistochemistry for GATA3.

Case	GATA3
**MGA**	
Case 1	0
Case 2	20 (1)
Case 3	0
Case 4	20 (1)
Case 5	85 (1)
Case 6	0
Case 7	15 (1)
Case 8	0
**Adjacent BC**	
Case 1	0
Case 2	0
Case 5	95 (1)
Case 7	15 (1)

Percentage of positive lesional cells. Staining intensity is given in parenthesis: 1 (weak).

## Discussion

MGA is a poorly understood lesion of the mammary gland. Initially, it was considered a benign tumor or reactive epithelial change [1, 2]. A small number of clinical and molecular studies on MGA has been published since its first description [2, 3, 4, 5, 6, 7, 8, 9, 10, 11, 12], and the biology of MGA is still little known [6]. The current study confirms and extends previous findings on the molecular pathology of MGA. We characterized a series of eight cases of MGA, four of which were lesions without adjacent invasive BC.

Consistent with the study of Shin *et al*, DNA CNAs were highly variable in MGA, ranging from almost no change to a highly complex pattern of chromosomal gains and losses [9]. CN gain on chromosome 2q was the most frequent CNA and was more common in our series than previously reported by Shin *et al* [9]. FISH analysis for the *ERBB4* gene locus (chromosome 2q34) confirmed CN gain on chromosome 2q in five of eight MGA cases. However, CN gain on chromosome 2q34 was not associated with enhanced ERBB4 protein expression, as determined by immunohistochemistry (data not shown).

In accordance with the studies of Guerini‐Rocco *et al* and Geyer *et al*, mutational characteristics were highly variable in MGA [11, 12]. Genes found to be mutated in MGA in previous studies and in our analyses included *TP53*, *PTEN*, *FGFR2*, *ATM*, *PIK3CA*, and *PMS2* [11, 12]. Newly discovered mutations in MGA affected the *CTNNB1*, *ARID1A*, *FBXW7*, and *MET* genes. Interestingly, *TP53* mutations were less frequent than previously reported. In the study of Guerini‐Rocco *et al*, 7 of 12 (58%) MGA cases harbored a *TP53* mutation [11]. These mutations were almost exclusively missense mutations, and MGA lesions were positive for p53 by immunohistochemistry [11]. In the present study, *TP53* mutations were detected in only two of seven (29%) MGA cases. Both mutations were truncating mutations, and none of the MGA lesions showed nuclear p53 accumulation by immunohistochemistry. We conclude that immunohistochemistry for p53 is of limited value for the histological differential diagnosis of MGA.

Consistent with the studies of Shin *et al*, Guerini‐Rocco *et al*, and Geyer *et al*, CNAs and mutational characteristics of BCs adjacent to MGA indicated clonal relatedness. On morphological grounds, a relationship between MGA and adjacent BC has already been described because both lesions may exhibit similar cytoplasmic clearing, a similar growth pattern, and a similar triple‐negative immunophenotype [5]. These morphological features were also encountered in all cases of the present series. It remains to be seen whether MGA can be considered the precursor lesion of concomitant invasive BC, as suggested by Guerini‐Rocco *et al*. Alternatively, MGA and invasive BC could originate from a common neoplastic stem cell through different paths of clonal evolution. Although we and others found considerable overlap between CNAs and mutational profiles in MGA lesions and adjacent BCs, there were also mutations restricted to either MGA or adjacent BC. Because our NGS panel included only a limited set of genes (*n* = 161), linear or parallel evolution of MGA and adjacent BC remains an open and still unsolved issue [6]. On purely histomorphological grounds, transitions between both lesions, favoring a linear evolution, could not be demonstrated in our series.

Neither histological nor molecular characteristics appeared to enable differentiation between MGA with and MGA without associated BC. As a consequence, when encountered in diagnostic core needle biopsies, it is reasonable to categorize MGA as a B3 lesion (uncertain malignant potential) [6]. Complete resection of the lesion may be required in order to exclude an adjacent BC not sampled by the core needle biopsy. Hence, MGA should not be mistakenly classified as a B2 lesion.

For the first time, DNA methylation patterns of MGA have been analyzed. Increased DNA methylation suggested an epigenetic inactivation of the *GATA3* gene in MGA. Subsequent immunohistochemical analyses confirmed that GATA3 protein expression is constantly reduced or lost in MGA, as well as in associated BCs. GATA3 is a critical transcription factor, which regulates the development of the skin and the mammary gland [21, 23, 24]. Its expression is required for the differentiation and maintenance of the normal mammary epithelium. More specifically, GATA3 is required for luminal differentiation and ER expression [21, 23, 24]. *GATA3* is also one of the most frequently mutated genes in BC. *GATA3* mutations are typically gain‐of‐function mutations and, in most instances, can be observed in ER‐positive BCs [26, 27]. Mutant GATA3 displays altered DNA‐binding activity and can induce transcriptional programs normally not induced by wild‐type GATA3 [27]. In mouse models, loss of GATA3 can promote tumorigenesis by inducing a differentiation block and an expansion of undifferentiated luminal progenitor cells [28, 29]. It is tempting to speculate that MGA is the morphological correlate of an uncontrolled expansion of luminal progenitor cells, which has so far only been described in genetically engineered mouse models.

In conclusion, we present evidence that MGA represents a mammary gland neoplasm of uncertain malignant potential, which is characterized by CN gain on chromosome 2q and loss of GATA3 protein expression in conjunction with epigenetic inactivation of the *GATA3* gene.

## Author contributions statement

MR, MC and HK designed the study and assessed histomorphological characteristics. JLvL, HS, JB and DS performed DNA CN profiling. SB and UL carried out mutational analysis. MR, HC, LR, ML and CW performed FISH analyses. SB, HS, IG and UL carried out DNA methylation profiling. All authors contributed to data collection and final data analysis.

## Supporting information


**Figure S1.** Immunophenotypic characteristics of MGA
**Figure S2.** Whole‐genome CNA profiles of MGA cases
**Figure S3.** Clonal relatedness of CN profiles in MGA and adjacent BC
**Figure S4.** Quantitative methylation analysis by bisulfite pyrosequencing of *GATA3* in MGA
**Figure S5.** Loss of GATA3 expression in MGA

**Table S1.** Antibodies used for immunohistochemistry
**Table S2.** List of gene targets in Oncomine Comprehensive Assay v3
**Table S3.** Characteristics of specimens for DNA methylation profiling
**Table S4.** Clinicopathological characteristics of carcinomas used for pyrosequencing assay
**Table S5.** *GATA3* pyrosequencing assay primers
**Table S6.** CN gains/losses in MGA and adjacent BC
**Table S7.** *ERBB4* CN by FISH
**Table S8.** NGS details
**Table S9.** List of genes examined in the 850 K array analysis
**Table S10.** CpG sites within *GATA3* examined in the 850 K array analysis
