# Supplementary material for: Chromosome 2q gain and epigenetic silencing of GATA3 in microglandular adenosis of the breast
Source: J Pathol Clin Res. 2020 Dec 31;7(3):220–32. doi: 10.1002/cjp2.195 (PMC8073017; doi:10.1002/cjp2.195)
Supplement: Supplementary file 1 — Figure S1. Immunophenotypic characteristics of MGA Figure S2. Whole‐genome CNA profiles of MGA cases Figure S3. Clonal relatedness of CN profiles in MGA and adjacent BC Figure S4. Quantitative methylation analysis by bisulfite pyrosequencing of GATA3 in MGA Figure S5. Loss of GATA3 expression in MGA [file CJP2-7-220-s001.docx]

**Chromosome 2q gain and epigenetic silencing of GATA3 in microglandular adenosis of the breast**

M Radner *et al*. *J Pathol Clin Res* DOI: 10.1002/cjp2.195

**Supplementary Figures S1 – S5**

**
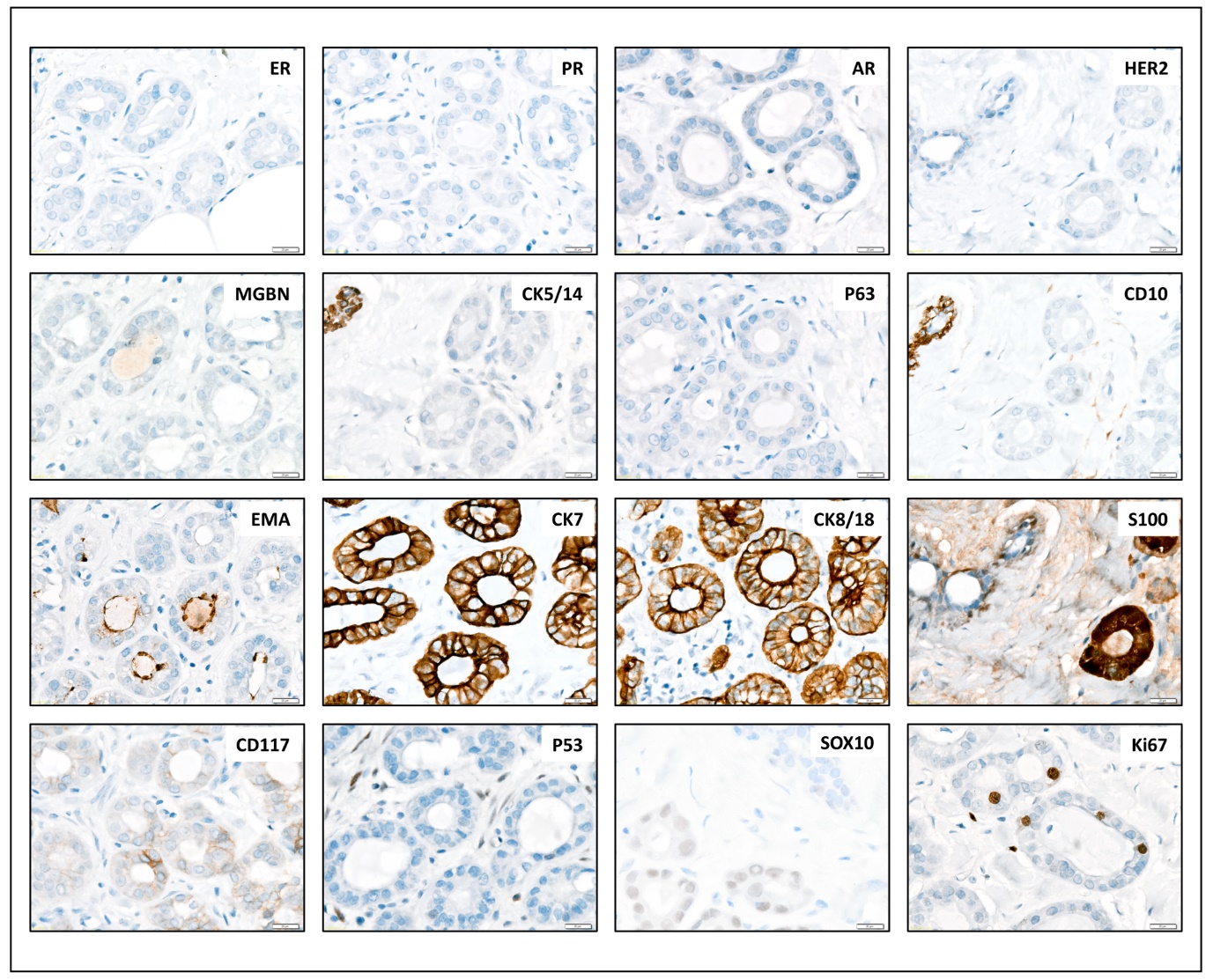
**

**Figure S1.** Immunophenotypic characteristics of MGA.

Representative immunohistochemical photomicrographs of MGA (case 1) showing the typical ER-, PR- and HER2-negative phenotype and strong expression of S100. Negative immunostaining for CK5/14, p63 and CD10 indicates the absence of a myoepithelial cell layer.

**
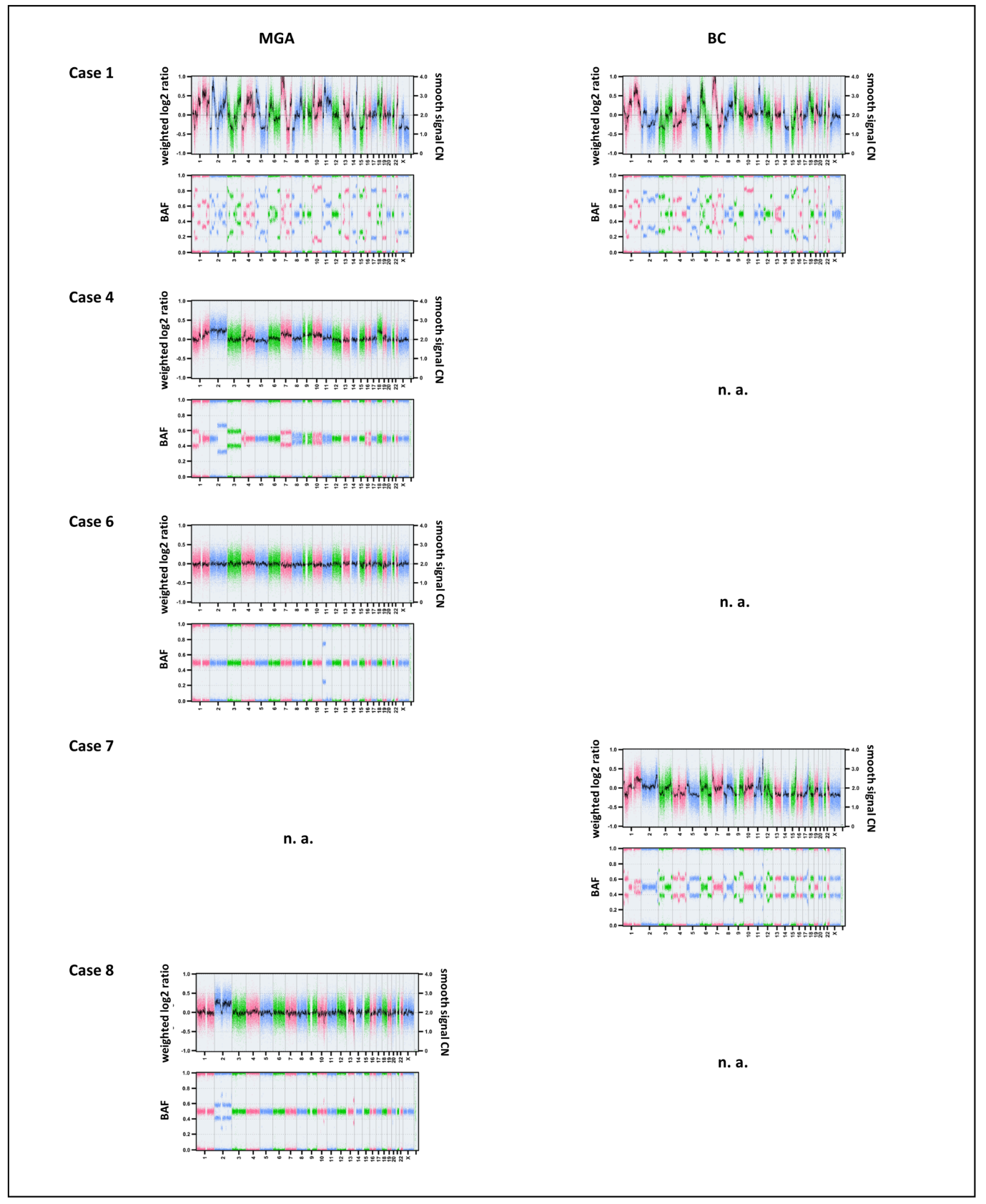
**

**Figure S2.** Whole genome CNA profiles of MGA cases.

Shown are the whole genome CNA profiles of cases 1, 4, 6, 7 and 8. CNA profiles of MGA lesions are on the left, CNA profiles of the adjacent BCs are on the right side. The upper plots show weighted log2-ratios and copy numbers (represented as a Gaussian smoothed calibrated copy number estimate) on the left and right *y*‐axis, respectively. Chromosomal localization is represented on the *x*-axis. The lower plots show the corresponding B-allele frequency (BAF). Gains of chromosome 2q can be seen in both MGA cases and the adjacent BCs. Only case 6 does not show a gain in chromosome 2.

**
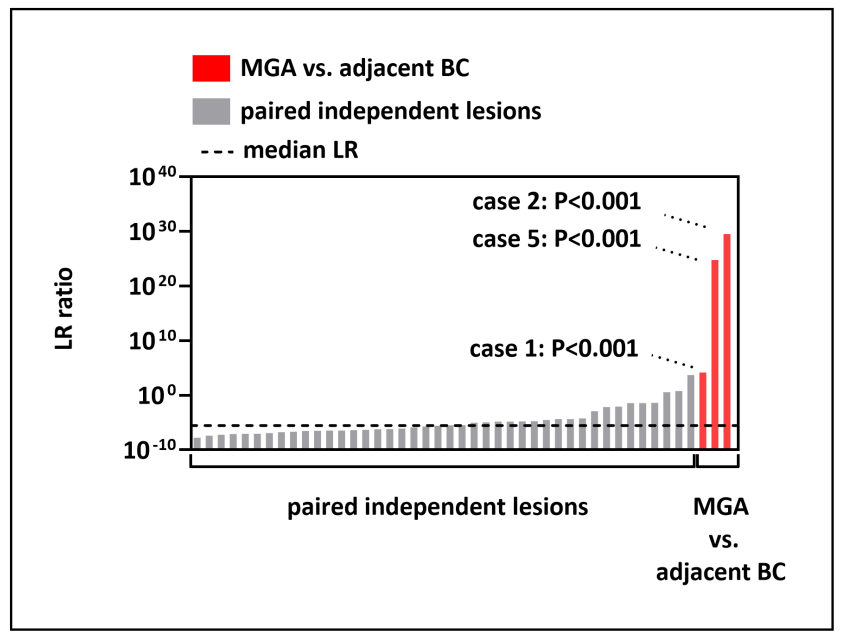
**

**Figure S3.** Clonal relatedness of CN profiles in MGA and adjacent BC.

The diagram shows the statistical likelihood ratio (LR) on the *y*-axis. Represented on the *x*-axis are the pairs of clonal and non-clonal lesions, respectively. Each bar indicates the paired CN profiles of two lesions providing n=42 non-clonal lesion pairs from independent patients (gray) and n=3 clonal lesion pairs from MGA and adjacent BC (red; case 1, 2 and 5). The LRs for MGAs and adjacent invasive BCs ranged from 1.5x10^4^ to 3.0x10^29^, which formally proved clonal relatedness of adjacent lesions in individual patients (3x P<0.001).

**
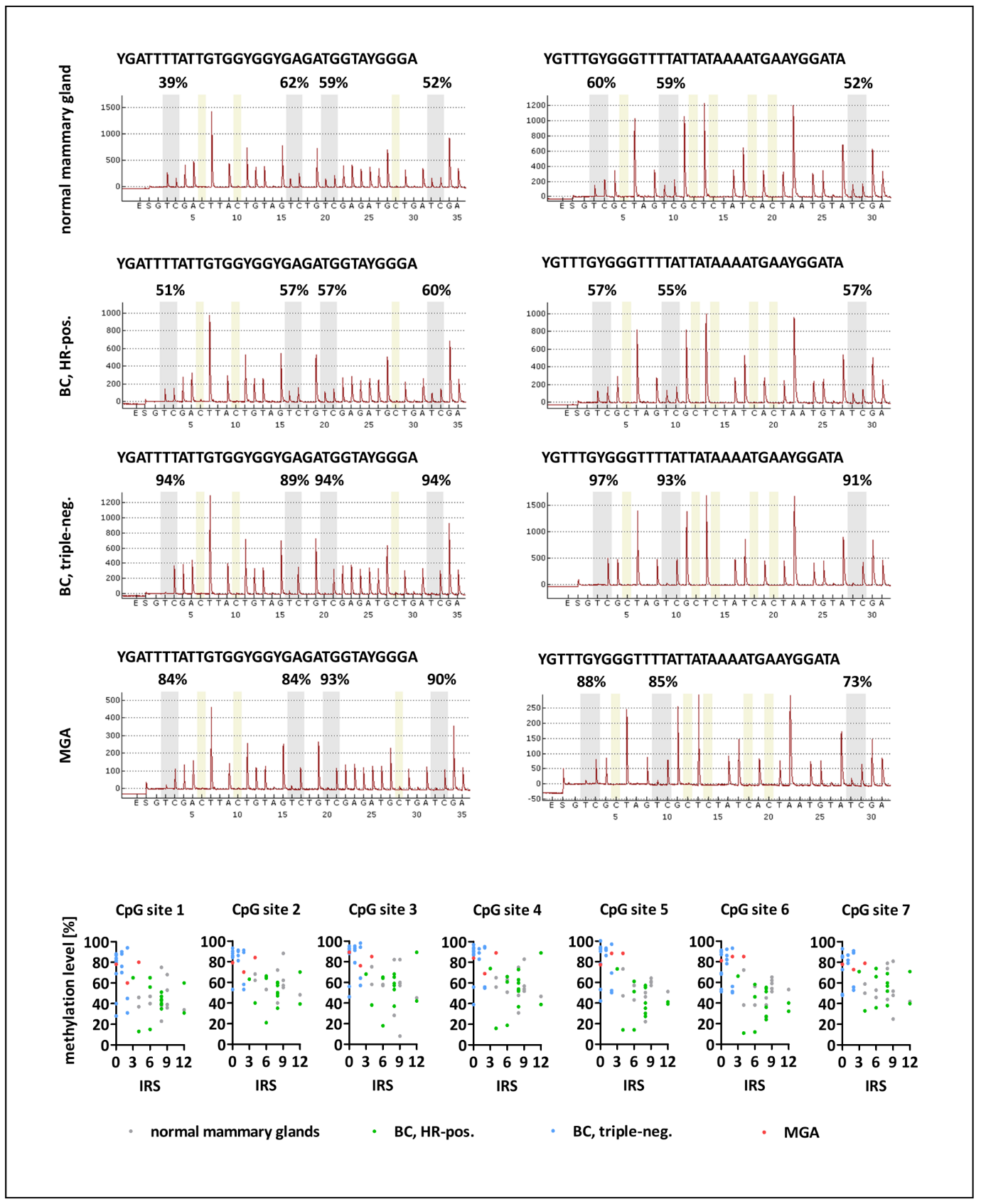
**

**Figure S4.** Quantitative methylation analysis by bisulfite pyrosequencing of *GATA3* in MGA.

The pyrograms show seven individual CpG sites in exon 4 of *GATA3* in which MGA lesions presented significantly higher DNA methylation levels than normal mammary tissue or HR-positive BCs while triple-negative BCs resemble MGA. The scatter plots below for each CpG site show the methylation levels (*y*-axis) compared to the IRS (immunoreactive score; *x*-axis) for GATA3 of each specimen in one of the four examined tissue types. Note the high methylation levels of *GATA3* and the missing or reduced immunohistochemical expression of GATA3 in MGA and triple-negative BC. Red dots represent MGA, green dots represent HR-positive BC, blue dots represent triple-negative BC and gray dots represent normal mammary glands. MGA lesions included n=3 cases (cases 1, 4 and 5). Normal mammary specimens (n=13), HR-positive BCs (n=14) and triple-negative BCs (n=18).

**
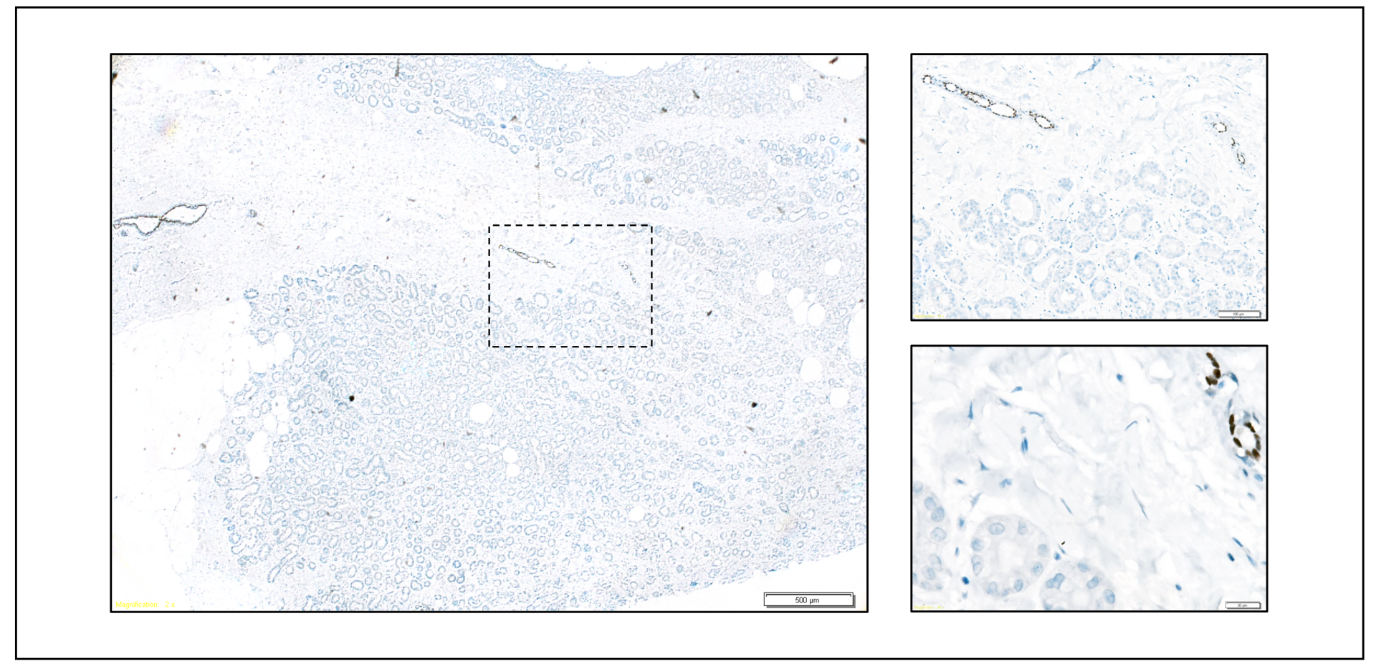
**

**Figure S5.** Loss of GATA3 expression in MGA.

The glandular formations of this representative MGA (case 1) show the absence of GATA3. The dashed box highlights a normal duct with intraluminal GATA3-positive cells which serve as an internal positive control.
